# Scattering of Light from the Systemic Circulatory System

**DOI:** 10.3390/diagnostics10121026

**Published:** 2020-11-30

**Authors:** Sidra Batool, Mehwish Nisar, Fabio Mangini, Fabrizio Frezza, Eugenio Fazio

**Affiliations:** 1 Department of Information Engineering, Electronics and Telecommunications, Sapienza University of Rome, Via Eudossiana 18, 00184 Rome, Italy; mehwish.nisar@uniroma1.it (M.N.); fabrizio.frezza@uniroma1.it (F.F.); 2 Department of Fundamental and Applied Sciences for Engineering, Sapienza University of Rome, Via A. Scarpa 16, 00161 Rome, Italy; eugenio.fazio@uniroma1.it; 3 Department of Information Engineering, University of Brescia, Via Branze 59, 25123 Brescia, Italy; fabio.mangini@uniroma1.it

**Keywords:** optical properties, scattering theories, circulatory system

## Abstract

There are many factors of methodological origin that influence the measurement of optical properties of the entire circulatory system which consists of blood as the basic component. The basic idea of this review article is to provide the optical properties of the circulatory system with all those factors of influence that have been employed in biomedical optics for different applications. We begin with the available optical properties, i.e., absorption, scattering and, reduced scattering coefficient, in general for any tissue inside the human body and prominent scattering theories (e.g., light, X-rays, neutrons) that are helpful in this regard. We have reviewed and compiled already available formulas and their respective available data for different human tissues for these optical properties. Then we have descended to the blood composition and to different scattering techniques available in the literature to study scattering and light propagation inside blood. We have reviewed both computational and theoretical scattering techniques.

## 1. Introduction

When light interacts with the human body, several phenomena take place depending on the type of tissue and on its biological composition [1], such as reflection, refraction, absorption, scattering or even emission. The different optical properties of the tissues influence the interaction with light and consequently the various diagnostic and therapeutic applications. The heterogeneity of the tissues increases the complexity of the analysis of interactions with light; therefore, following a methodological approach for further study, it is useful to first analyse the factors that influence the average optical properties on a macroscopic scale and then deal with the microscopic effects related to the heterogeneity of the individual structures. The optical properties that mainly describe the transport of light in the tissues are absorption and scattering. The absorption of light is due to the electronic transitions that can occur in the molecules that make up the tissues and whose selectivity, consequently, gives colour to the tissues (often the coloration of the fabric is identified with that part of the molecule responsible for absorption called chromophore, a word coming from the Greek term “chroma” which means colour) [2,3,4,5]. Electronic transitions typically have energies of the order of electronvolts and therefore occur in the optical band, i.e., partly in the UV, in the visible and partly in the IR too where the molecular transitions (rotations/vibrations/roto-vibrations) have more their characteristic energies. The scattering of light is instead due to fluctuations and discontinuities in the refractive index of the optical tissue due to the presence of cells, membranes, microstructures and sub-cellular components [6]. The most used technique for the characterization of the optical properties of matter is optical spectroscopy, with which it is possible to determine the light absorption and emission absorption properties. It is possible to carry out both absorption spectroscopy, revealing the spectral components of the sent light which have been absorbed or diffused due to absorption or scattering, and emission spectroscopy, in which case, given a high energy excitation, the spectral components of the radiation emitted by the material are measured [7,8,9,10]. In the next section, we shall describe the scattering and absorption of light by biological tissues.

## 2. Fundamental Optical Properties of Tissues

**Figure 1:**
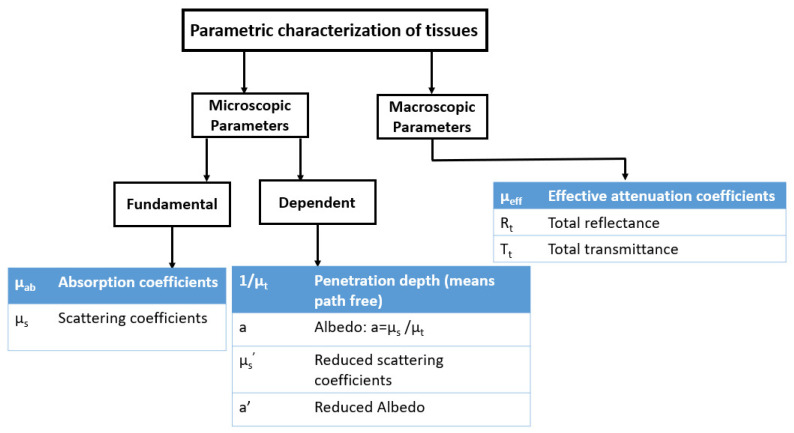
Parametric characterization of tissues on microscopic and macroscopic levels [27].

Tissues are generally characterized by structural inhomogeneities of very different dimensions, from tenths of nanometers to hundreds of micrometers [11]. For this reason, many different techniques are used for the microscopic and macroscopic characterization of the optical properties of the tissues, as shown in Figure 1 [12,13,14,15], and many different simulation models have been implemented to highlight the specific behaviour of the materials and the propagation of the light inside them. The light scattering from spherical particles is mainly described by the Rayleigh and Mie theories. The Rayleigh scattering indicates scattering by small particles or mass density variation much smaller than the wavelength of light, while the Mie scattering indicates the scattering by particles comparable to or larger than the wavelength of light [16,17,18,19,20,21,22]. One of the simplest and most used models for light scattering by biological tissues implements a Mie scattering procedure. Such a model represents a biological tissue as a mixture of particles of varying sizes dispersed within a host matrix with a different refractive index [23,24]. Mie scattering is defined as when the size of the object is larger than the wavelength of light. This type of model is certainly effective even, if the idealization of the sphericity of the scattering centers leads to results sometimes different from the real behaviours. An alternative approach to Mie scattering is represented by the continuum model, in which a tissue is modeled as a continuous medium where there are fluctuations in density and therefore in the refractive index, which can be described through an autocorrelation function [25]. The Wiener–Khintchine theorem, in general, relates the autocorrelation function to the corresponding spectrum of the scattered power [26].

The popular scattering theories that have been employed in many fields of biomedical optics are Rayleigh and Mie scattering theories. The Rayleigh scattering indicates scattering by small particles or mass density variation much smaller than the wavelength of light, and Mie scattering indicates the scattering by particles comparable to or larger than the wavelength of light.

### 2.1. Absorption Coefficient

The absorption of light by an optimum volume of identical molecules can be expressed in terms of the absorption coefficient, which represents the probability that a photon is absorbed with respect to the medium according to its required path-length. Let us consider a homogeneous medium composed by absorbing molecules. The absorption coefficient is defined as the product between the probability that a photon is absorbed by a molecule (i.e., the cross-section *a*) and the density of the molecules present in the volume:

(1) $$\mu_{a}=\rho_{a}\sigma_{a}$$

The absorption cross section measures the rate at which the photon energy is being absorbed from the light wave [27].

### 2.2. Scattering Coefficient

Similarly to absorption, the elastic scattering for single events (i.e., not for multiple scattering processes) in a certain medium can still be represented by a scattering coefficient *s*, that describes the probability of photon to be scattered during a unitary path-length. The reciprocal of *s* is called the scattering length of the object and denoted as the average distance traveled by a photon between consecutive scattering events (photon mean-free-path). Furthermore, the scattering coefficient may be defined as [28] as the product between the probability that a photon is diffused by a scattering center (i.e., the cross section *s*) and the density *s* of scatterers in the volume:

(2) $$\begin{array}{c} \mu_{s}=\rho_{s}\sigma_{s} \end{array}$$

**Figure 2:**
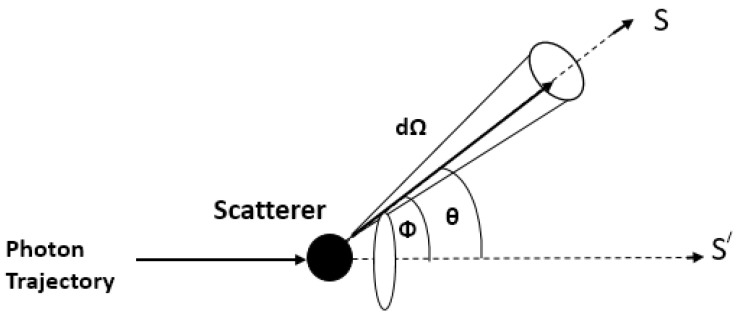
A photon initially traveling from $S^{\prime }$ is scattered into a differential solid angle dΩ along *S*. Here ϕ corresponds to the azimuthal angle from the scattered trajectory, and θ corresponds to the polar angle from the initial trajectory.

The scattering cross-section area is defined as the rate with which the power is removed from the initial $S^{\prime }$ direction towards the new *S* direction compatible with the medium, as shown in Figure 2. The probability that a photon is scattered within a certain solid angle is defined by the phase function *p*:

(3) $$\begin{array}{c} p=\frac{1}{\sigma_{s}}\frac{d\sigma_{s}}{d\Omega } \end{array}$$

where the term $\frac{d\sigma_{s}}{d\Omega }$ represents the differential scattering cross section [29]. Both the scattering and the absorption coefficients depend on the light wavelength [30,31].

### 2.3. Reduced Scattering Coefficient

Instead of using the whole scattering theories, often an equivalent reduced scattering coefficient is used to describe both Mie and Rayleigh regimes. This reduced coefficient can effectively describe the dispersion properties of the main six different groups of tissues such as skin, breast, brain, bones, fibrous and fatty tissues. All of them are a set of small and large scatterers that act simultaneously. Its expression refers to the scattering efficiency at a reference visible wavelength 0 (usually the green @500 nm) according to the following wavelength-dependent expression:

(4) $$\begin{array}{c} \mu_{s}^{\prime }=a{\left(\frac{\lambda }{500\;(\mathrm{nm})}\right)}^{-b} \end{array}$$

where *a* is the $\mu_{s}{}^{\prime }$ value at the reference wavelength and *b* is the characteristic power dependence. A more explicit expression of the scattering coefficient separates the Rayleigh and the Mie contributions keeping the same wavelength-type dependence:

(5) $$\begin{array}{c} \mu_{s}^{\prime }=a^{\prime }\left(f_{Ray}{\left(\frac{\lambda }{500\;\mathrm{nm}}\right)}^{-4}+\left(1-f_{Ray}\right){\left(\frac{\lambda }{500\;\mathrm{nm}}\right)}^{-b_{Mie}}\right) \end{array}$$

where $f_{Ray}$ and $f_{Mie}$ represent the fraction of small and large scatterers, respectively and satisfy the conservation rule:

(6) $$\begin{array}{c} f_{Ray}+f_{Mie}=1 \end{array}$$

**Figure 3:**
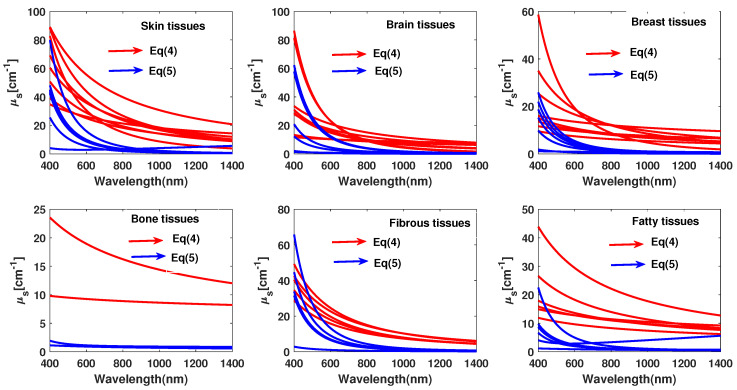
In the review literature, some measurement expressing the optical properties of tissues [27].

In Figure 3 the explicit expressions and values of $\mu_{s}^{{}^{\prime }}$ are reported for different tissues. All of them, as shown by the previous analytical expressions illustrated in Table 1 and Table 2, have a hyperbolic decreasing dispersion increasing the wavelength, in the VIS-NIR spectral range [32,33,34,35].

## 3. Scattering of Light from Nerves, Veins, and Arteries

The polarization of light contributes to the observed appearance of the skin and its relative colour, since the reflection/refraction/diffraction of the light on it depends strictly on the degree of polarization of the light used. Since on some chromatic bands the absorption is very high and on others much less, the observed effects can be either superficial or deep, with light penetrations in the underlying layers either limited to a few microns or extended to several centimeters [50,51,52].

Based on conventional tissue staining protocols, pathological understanding of arterial diseases is mainly attributable to observations that have been reported in the literature [53]. A new venue for visualizing pathological changes in the extracellular matrix caused by the progression of atherosclerosis is the emerging development of Nonlinear Optical Microscopy (NLOM), especially in second-harmonic generation, two-photon excited fluorescence and, coherent Raman scattering. In general, these techniques can offer rapid three-dimensional imaging in the biomedical sector. In this review, we look at recent progress in applications related to arterial disease imaging using various forms of NLOM [53,54].

**Figure 4:**
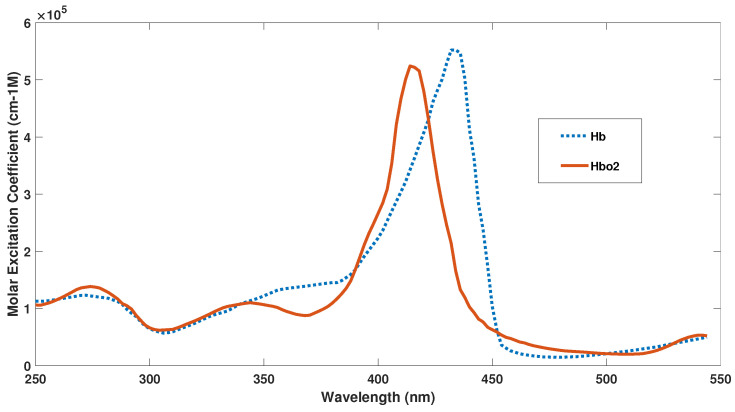
The graph shows the best-estimated spectrum of Hb and $HbO_{2}$ from the various sources by Scott Prahl Data [57].

During internal propagation, the light undergoes both scattering, due to the strong inhomogeneity of the biological tissue (formed by dispersed micro and nanostructures, filamentous proteins, cells, fibres, successive layers of dermis, fat, veins and capillaries, etc.), and absorption, due to the presence of many chromophores (the main ones being haemoglobin and melanin [39,55,56]. The melanin function is essentially the absorption of light radiation on some spectral bands, for example, the erythemal UV between 290 and 320 nm, which could be harmful to the body). Through the skin, the veins are also visible and with them the blood circulating inside, being able to make remote measurements in a non-invasive and non-bloody way. Focusing attention on it, the absorption spectrum of oxygenated (*HbO*${}_{2}$) and deoxygenated haemoglobin (*Hb*) is shown in Figure 4 [57].

### 3.1. Haemoglobin Absorption

As you can see, the two forms, Hb and ${\mathrm{HbO}}_{2}$, have absorption peaks posted with respect to each other. Therefore, by making differential absorption measurements on the different peaks, it is possible to control the level of oxygenation with great precision [58]. This different absorption also influences the observed colour of the blood: oxygenated blood, within arteries, has a cherry red colour while the deoxygenated blood, present in the veins, is dark red (but through the skin it looks blue). The absorption rate of light is higher in the blue band than in the red band, because dark red deoxygenated venous blood has a higher absorption coefficient than cherry red oxygenated arterial blood in the human body [59]. However, the arteries are generally brighter than the veins. This is because they have thick vessel walls as they have to withstand high pressures due to heart beating. The arterial walls are typical of elastic and sometimes muscular tissues, which dilate and contract both to withstand the large pressures induced by heart pulses and to regulate and control the resistance of the flow in the capillary network. The veins, on the other hand, are used only for the transport of low oxygen blood to the organs used for its purification, lungs, and liver. For this reason, venous blood appears very dark to the eye [60,61]. Arteries and veins have different dispositions within the human body too: the arteries travel deep and are difficult to see and not accessible from the outside; the veins, on the other hand, are typically superficial and therefore can be easily monitored with non-invasive imaging techniques. However, it is possible to make non-bloody and non-invasive measurements also on the arteries by monitoring the retinal one: in fact, in the retina, there is a network of arterial capillaries between cones and rods of the retina. The central artery of the retina, through the dural sheath of the optic nerve, reaches the optic papilla, where it divides first into an ascending and descending branch and then, subsequently, into lateral and medial branches. In this way, a dense network of small vessels is formed that never anastomose and that is easily accessible for observation by intraocular retinal imaging techniques. For many medical applications, it is of great importance to measure blood pressure and check for abnormal changes in veins and arteries that could be analyzed in depth using these retinal imaging techniques. In fact, many diseases cause an abnormal ratio between the length, diameter, and cross-linking of arteries and veins. For example, in diabetic patients the veins are abnormally wide, while pancreatic diseases lead to a narrowing of the wall of the vessels which generates an increase in blood pressure [5,62]. Minhaj et al. have shown that by combining together the optical density ratio (ODR) and the tracking of blood vessels (BVT) it is possible to make and characterize the map of the vessels, both arteries and veins. This is often traced starting from the source nodes and uses the curvature and the angle to derive the whole network. This measurement technique, despite being qualitative, has an accuracy of 97.06% in identifying veins and arteries [63]. Furthermore, the different response of oxygenated and deoxygenated hemoglobin to electromagnetic excitation finds application in functional magnetic resonance [64].

### 3.2. Optical Properties of Human Blood and Its Composition

Normal human blood consists of red blood cells (RBCs or erythrocytes, ±4500 × 103/μL blood), white blood cells (leukocytes, ±8 × 103/μL blood), platelets (thrombocytes, ±300 × 103/μL blood) and blood plasma that mainly contains water, electrolytes, plasma proteins, carbohydrates, lipids and various extracellular vesicles. The major portion of RBCs consists of haemoglobin. In healthy human adults, the haemoglobin concentration in blood is 140 g/L in women and 155 g/L in men on average.

**Figure 5:**
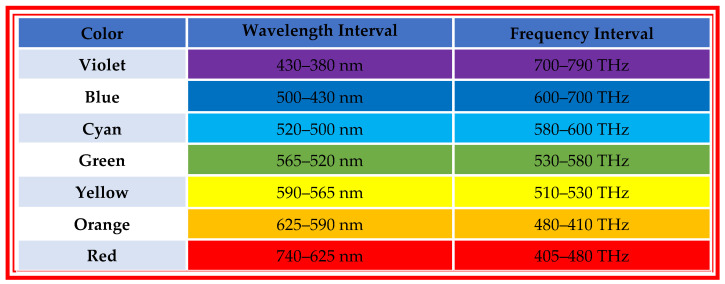
White light spectrum with its corresponding wavelength and frequency interval.

Scattering and absorption of light in the blood is largely conducted by red blood cells for many reasons. Firstly, it should be remembered, that the quantity of red blood cells is about 4000 times greater, than that of white blood cells and therefore, they constitute the largest mass fraction. In humans, red blood cells have the typical shape of flexible biconcave discs, 6–8 μm in diameter, and 2.5 μm thick [58,65,66]. They dominate blood light scattering over any other component of the blood both for their large size and for the contrast index of refraction between the red blood cells and the surrounding blood plasma. In fact, their refractive index has a value of approximately between 1.40 and 1.42 [67], due to the combination of ${\mathrm{HbO}}_{2}$ (1.615) and water (1.333) [68], while the refractive index of plasma (and serum) ranges from 1.36 (@400 nm) to 1.34 (@800 nm) [69]. Absorption is instead dominated by hemoglobin, in the two deoxygenated Hb and oxygenated ${\mathrm{HbO}}_{2}$ forms (Figure 5). Flow cytometric light scattering measurements are helpful to determine the concentration of RBCs and their volume fraction, the hemoglobin concentration, and to discriminate the health of blood cells, as for example thalassaemic vs normal RBCs [70,71,72,73].

## 4. Computational Techniques in Literature

Many numerical models are used to describe the propagation of light within diffusive environments such as for example liquid suspensions of red blood cells. It is the most used, the Monte Carlo (MC) method is one of the most used statistical methods to describe multivariable dynamic systems. It is based on the dynamic simulation of the propagation of a population of elements which in our case are photons. By imposing the rules of absorption and diffusion of light passing through a suspension of RBCs, the MC method analyses in parallel the propagation of a set of photons statistically independent through the suspension. At each iteration a single photon may (1) not suffer anything and continue straight or (2) be absorbed or (3) be diffused. Statistically reconstructing the evolution of the entire population is initially possible to estimate the behaviour of light when passing through an RBCs suspension. The MC method is accessible to record the transmission and reflection coefficients from the tissues; from a comparison of the numerical results with the experimental data, it is possible to identify the micro topologies developed, the succession of the tissue layers, the properties of the materials, and the efficiencies of absorption and scattering [74,75].

The finite differences in time domain (FDTD) is a method of solving the electromagnetic wave equation using a step integration (finite differences) in the time domain. The method, in principle conceptually simple, is based on the definition of a derivative as a ratio of finite and not infinitesimal increments. This approximation is very delicate and leads to converging results if the rules defined by the Nyquist–Shannon sampling theorem [in the book Advances in FDTD Computational Electrodynamics] are respected [76]. Subsequent versions of the model have been developed over the years to decrease the error resulting from sampling using more complex definitions of the derivative as a ratio of symmetrical increments with respect to the calculation point. The FDTD method is general and flexible in terms of implementation of the full-wave techniques used to solve complex biological scattering problems [77]. It is suitable for the numerical simulation of scattering from non-homogeneous objects of arbitrary forms, as it numerically solves Maxwell’s equations and it can calculate the angular dispersion of the scattered light [78,79]. For readers interested in the FDTD method, the book by Taflove [76] is recommended for further information. An effective method of optimizing the scattering results produced by FDTD simulations takes advantage of the Rytov approximation, and is often applied in tomography. Starting from the phase mapping of the scattered light and comparing it with the experimental data, the method exercises an iterative process of convergence to optimize the distribution of the scattering centers [80,81]. The Discrete Dipole Approximation (DDA) is closely related to the method of moments applicable for multiple scattering from RBCs. The working principle of this method is based on the decomposition of the volume into infinitesimal elements, each represented as a dipole. The electromagnetic field is calculated as the superposition of the injected field with the secondary ones emitted by the induced dipole sources [82]. Jiangping et al. performed a comparison of these methods, finding that the Rytov approximation is less accurate than DDA and FDTD methods [80].

## 5. Theoretical and Experimental Techniques in the Literature

There has been a considerable amount of consequence in applying polarization properties of light to biomedical imaging usage, in the recent years. Optical polarization imaging is examining to be a popular convenient non-invasive technique for the detection of biological structures. The ultimate goal is the detection of millimeter-sized small tissues in the biological texture. A few of the research that used polarization principles for biomedical imaging were expressed in the following section. Demos et al. reported that the techniques regarding polarization principles for non-invasive surface imaging of biological systems [83,84]. Polarization discrimination of the scattered photons were precisely used employing a non-rotating retarder polarimetric configuration to enhance the visualness of the subsurface textures. Jacques et al. [85] used the simple polarization principle for visualizing superficial layers of tissue such as skin, breast, brain, bones, connective tissue and fat. The study gives the detailed information on the transition of linearly polarized light into randomly polarized light using the light propagation through tissues. Results from the study demonstrated that the polarized light imaging of skin yields images based on backscattered photon from apparent epidermal and initial papillary dermis however, the birefringent dermal collagen immediately irregular polarized light.

Sankaran et al. [86] described the light propagates through tissue phantoms with densely packed scatterers show the changes in the properties of polarization. To study the increase and decrease in linear and circular polarization were used polystyrene microspheres suspended in an aqueous solution. This study was playing a vital role in designing and optimizing polarimetric techniques for tissue imaging and diagnostics. Shamaraj et al. [87,88] reported the study of scattered polarized light from highly scattering media are helpful for an analytical model and material characterization techniques. This study might be described in understanding the techniques for imaging tissues and hidden objects, which can be, used for the treatment of malignant diseases and diagnostics. Kari et al. [89] investigated the use of polarized light reflections to image tissues such as wood fibers. The components of the reflected light from the wood fibers contained information regarding the structure of the tissue. They described the wood-fiber absorption, scattering cross sections, and scattering matrices in the ray-optics approximation. They computed the complicated internal structure of the wood fibers.

### 5.1. Fundamentals of Polarized Light Scattering

**Figure 6:**
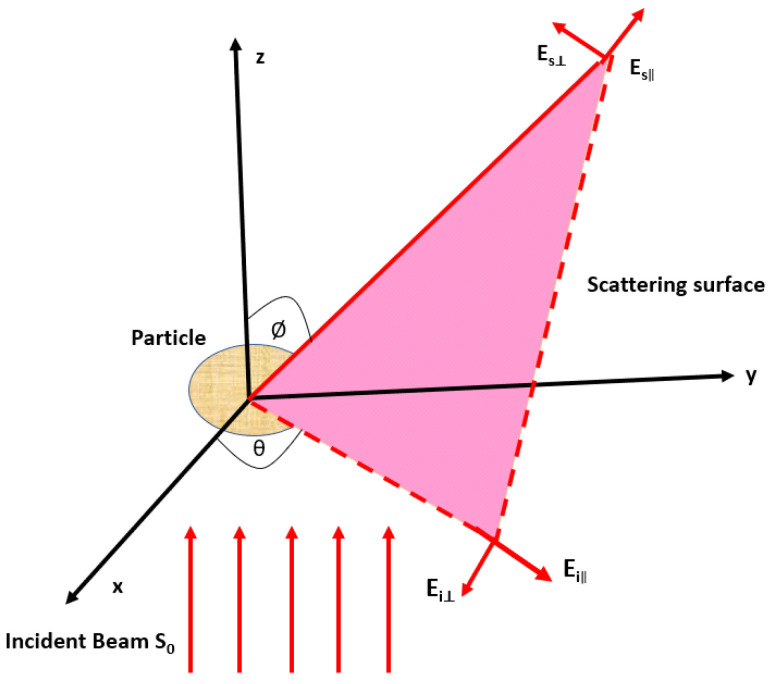
Geometry represents the scattering of light by an object located at the origin.

An incident of polarized light on an object can be described as two orthogonal linear polarization components of the incident light field parallel $E_{i||}$ and perpendicular $E_{i⊥}$ to the scattering plane shown in Figure 6.

This figure represents the geometry of the scattering of light by an object (a particle representing the primary components of a biological structure). Here, the incident light beam $S_{0}$ is parallel to the z−axis, θ and ϕ are the scattering angles in the scattering plane and in the plane perpendicular to the scattering plane, respectively. The detecting plane located at a distance r from the origin along the vector $S_{1}$. two orthogonal polarization components, $E_{s⊥}$ and $E_{s||}$ of the scattered light formed a specific state of polarization depending on the amplitudes and phase shifts between components. The linear relationship between incident and scattered components can be described by the transformation of an arbitrarily polarized light (linear, circular, or elliptical) [90,91].

(7) $$\begin{array}{c} \left[\begin{array}{c} E_{s||}\\ E_{s⊥} \end{array}\right]=\frac{e^{-ik\left(r-z\right)}}{-ikr}\left[\begin{array}{cc} S_{1} & S_{2}\\ S_{3} & S_{4} \end{array}\right]\left[\begin{array}{c} E_{i||}\\ E_{i⊥} \end{array}\right] \end{array}$$

where $k=\frac{2\pi }{\lambda }$ is a wave number, $\iota =\sqrt{-1}$, r represents the distance from the scatterer to the detector, *z* is the position coordinate of the scatterer. The matrix elements from $S_{1}$ to $S_{4}$ are described the amplitude of the scattering matrix (S-matrix) or Jones matrix. Each element depends on an elevation and azimuthal angles θ and ϕ, which contain information regarding the scatterer. Both phase and amplitude must be measured to evaluate the amplitude scattering matrix. The Jones matrix elements of transparent optical materials without sign ambiguity can be determined by using polarimetric methods.

### 5.2. Polarimetric Imaging

Polarimetry is the technique of measuring polarization state and interaction of polarized light with the target object always returns the change in the state of polarization of the interacting light. Weakly scattered light retains its polarization state easily, meanwhile strongly scattered light does not retain its polarization state [92]. Investigating the state of polarization of light interaction with the target results in a better imaging procedure to extract useful information from the target.

### 5.3. Stokes Parameter

The polarization state of light can be adequately demonstrated by the four Stokes parameters as shown below.

(8) $$\begin{array}{cc} \; & I=E_{x}^{2}+E_{y}^{2} \end{array}$$

(9) $$\begin{array}{cc} \; & Q=E_{x}^{2}-E_{y}^{2} \end{array}$$

(10) $$\begin{array}{cc} \; & U=2E_{x}E_{y}\cos\delta \end{array}$$

(11) $$\begin{array}{cc} \; & V=2E_{x}E_{y}\sin\delta \end{array}$$

where $E_{x}$ and $E_{y}$ describes the maximum amplitudes of the optical components of the field in *x* and *y* directions, respectively, and δ describes the phase difference between the optical components of the field.

I expresses the total intensity of light;

Q expresses the amount of linear horizontal or vertical polarization;

U expresses the amount of linear +45${}^{\circ }$ or $-45^{\circ }$ polarization;

V expresses the amount of right or left circular polarization contained in the light beam. The Stokes matrix may be written as:

(12) $$\begin{array}{c} S=\left[\begin{array}{c} I\\ Q\\ U\\ V \end{array}\right]=\left[\begin{array}{c} I_{H}+I_{V}\\ I_{H}-I_{V}\\ I_{P}-I_{M}\\ I_{R}-I_{L} \end{array}\right] \end{array}$$

where $I_{H}$ and $I_{V}$ are light intensities measured with horizontally and vertically oriented linear polarizers, $I_{P}$ and $I_{M}$ are measured with $+45^{\circ }$ and $-45^{\circ }$ oriented linear polarizers, $I_{R}$ and $I_{L}$ are measured with right circularly and left circularly oriented linear polarizers in front of a detector, respectively.

### 5.4. Mueller Matrix

When light comes in contact with the object, the polarization state of the light beam changes with respect to the medium. Eventually, the incident and exciting beam will have a different state of polarized light. The polarization of the scattered light from an object in far field shown in Figure 1. is investigated the Stokes vector $S_{s}$ associated with the Stokes vector of the incident light $S_{i}$ connected by the matrix equation $S_{s}$ = M $S_{i}$, where M is the normalized scattering matrix (Mueller matrix) [93,94].

(13) $$\begin{array}{c} \left[\begin{array}{c} I_{s}\\ Q_{s}\\ U_{s}\\ V_{s} \end{array}\right]=\left[\begin{array}{cccc} M_{11} & M_{12} & M_{13} & M_{14}\\ M_{21} & M_{22} & M_{23} & M_{24}\\ M_{31} & M_{32} & M_{33} & M_{34}\\ M_{41} & M_{42} & M_{43} & M_{44} \end{array}\right]\left[\begin{array}{c} I_{i}\\ Q_{i}\\ U_{i}\\ V_{i} \end{array}\right] \end{array}$$

Elements of the Mueller matrix depends on the scattering angle the wavelength of light, and the optical parameters of the object with the corresponding geometries. Element $M_{11}$ tell us the information of the incident light is unpolarized, and scattered light is a function of the moving angles, it allows long-range structure rather than the other light scattering matrix elements. Element $M_{12}$ is access by measuring the total scattered field intensity for a horizontally linearly polarized incoming light and subtracting the total scattered field intensity for a vertically linearly polarized incoming light. Element $M_{22}$ refers to the ratio of depolarized light to the total scattered light. $M_{34}$ refers to the transformation of the 45${}^{\circ }$ obliquely polarized incident light to circularly polarized scattered light. The elements $M_{33}$ and $M_{44}$ also displays the accurate measurement of the scattering beam from the object [95,96,97].

## 6. Conclusions

In this article, we have reviewed and compiled the optical properties of human tissues and the circulatory system with the main focus on blood. From this literature review, one of the main conclusions is that there are numerous factors of physical and methodological origin that are responsible for the study of the optical properties that anyone should aware of before performing own optical properties measurements. We have pointed out the main factors that influence absorption spectra of whole blood and hence influence optical properties. Revision of available polarimetric techniques can be helpful for the reader in the practice of biomedical optics. From this, we hope that we have provided the reader with a set of optical property spectra for whole blood and different polarimetric techniques that can be used in the practice of biomedical optics.

## Figures and Tables

**Table 1:** Represents the different tissues (e.g., skin, brain, breast, bone) description using scattering parameters, where a is the $\mu_{s}{}^{\prime }$ value at the reference wavelength and b is the characteristic power dependence and $f_{Ray}$ or $f_{Mie}$ demonstrated the fraction of small and large scatterers, respectively.

#	a	b	a${}^{\prime }$	$f_{\mathrm{Ray}}$	$f_{\mathrm{Mie}}$	Tissue Description	Ref
Skin							
1	48.9	1.548	45.6	0.22	1.184	skin	[36]
2	47.8	2.453	42.9	0.76	0.351	skin	[35]
3	37.2	1.39	42.6	0.4	0.919	skin	[37]
4	60.1	1.722	58.3	0.31	0.919	skin	[38]
5	29.7	0.705	36.4	0.48	0.22	skin	[39]
6	45.3	1.292	43.6	0.41	0.562	dermis	[40]
7	68.7	1.161	66.7	0.29	0.689	epidermis	[40]
Brain							
8	40.8	3.059	40.8	0	3.088	brain	[41]
9	10.9	0.334	13.3	0.36	0	cortex (frontal lobe)	[42]
10	11.6	0.601	15.7	0.53	0	cortex (temporal lobe)	[42]
11	20	1.629	29.1	0.81	0	astrocytoma of optic nerve	[42]
12	25.9	1.156	25.9	0	1.156	normal optic nerve	[42]
13	21.5	1.629	31	0.82	0	cerebellar white matter	[42]
14	41.8	3.254	41.8	0	3.254	medulloblastoma	[42]
15	21.4	1.2	21.4	0	1.2	brain	[25]
Breast							
16	31.8	2.741	31.8	0	2.741	breast	[41]
17	11.8	0.775	15.2	0.58	0	breast	[41]
18	24.8	1.544	24.8	0	1.544	breast	[41]
19	20.1	1.054	20.2	0.18	0.638	breast	[41]
20	14.6	0.41	18.1	0.41	0	breast	[43]
21	12.5	0.837	17.4	0.6	0.0076	breast	[44]
22	8.3	0.617	11.2	0.54	0.009	breast	[44]
23	10.5	0.464	10.5	0	0.473	breast	[45]
Bone							
24	9.5	0.141	9.7	0.04	0.116	skull	[42]
25	20.9	0.537	20.9	0	0.537	skull	[46]

**Table 2:** Represents the different tissues (e.g., fibrous and fatty) description using scattering parameters, where a is the $\mu_{s}{}^{\prime }$ value at the reference wavelength and b is the characteristic power dependence and $f_{Ray}$ or $f_{Mie}$ demonstrated the fraction of small and large scatterers, respectivelypectively.

#	a	b	a${}^{\prime }$	$f_{\mathrm{Ray}}$	$f_{\mathrm{Mie}}$	Tissue Discription	Ref
Fibrous tissue							
26	33.6	1.712	37.3	0.72	0	tumor	[41]
27	30.1	1.549	30.1	0.02	1.521	prostate	[47]
28	27.2	1.768	29.7	0.61	0.585	glandular breast	[48]
29	24.1	1.618	25.8	0.49	0.7845	fibrocystic breast	[48]
30	20.7	1.487	22.8	0.6	0.327	carcinoma breast	[48]
Fatty tissues							
31	13.7	o.385	14.7	0.16	0.250	Subcutaneous fat	[37]
32	10.6	0.520	11.2	0.29	0.089	Adipose breast	[48]
33	15.4	0.680	15.4	0.00	0.680	Subcutaneous adipose	[39]
34	35.2	0.988	34.2	0.26	0.567	Subcut. fat	[40]
35	21.6	0.930	21.1	0.17	0.651	Subcut. adipocytes	[40]
36	14.1	0.530	na	na	na	Adipose	[49]
